# Balancing novelty and appropriateness leads to creative associations in children

**DOI:** 10.1093/pnasnexus/pgac273

**Published:** 2022-12-02

**Authors:** Clara Rastelli, Antonino Greco, Nicola De Pisapia, Chiara Finocchiaro

**Affiliations:** Department of Psychology and Cognitive Science, University of Trento, 38068 Rovereto, Italy; MEG Center, University of Tübingen, 72076 Tübingen, Germany; Department of Neural Dynamics and Magnetoencephalography, Hertie Institute for Clinical Brain Research, University of Tübingen, 72076 Tübingen, Germany; MEG Center, University of Tübingen, 72076 Tübingen, Germany; Department of Neural Dynamics and Magnetoencephalography, Hertie Institute for Clinical Brain Research, University of Tübingen, 72076 Tübingen, Germany; Werner Reichardt Center for Integrative Neuroscience, University of Tübingen, 72076 Tübingen, Germany; Department of Psychology and Cognitive Science, University of Trento, 38068 Rovereto, Italy; Department of Psychology and Cognitive Science, University of Trento, 38068 Rovereto, Italy

**Keywords:** children, semantic search, creative problem solving, natural language processing, reinforcement learning

## Abstract

Creative problem solving is a fundamental skill of human cognition and is conceived as a search process whereby a novel and appropriate solution is generated. However, it is unclear whether children are able to balance novelty and appropriateness to generate creative solutions and what are the underlying computational mechanisms. Here, we asked children, ranging from 10 to 11 years old, to perform a word association task according to three instructions, which triggered a more appropriate (ordinary), novel (random), or balanced (creative) response. Results revealed that children exhibited greater cognitive flexibility in the creative condition compared to the control conditions, as revealed by the structure and resiliency of the semantic networks. Moreover, responses’ word embeddings extracted from pretrained deep neural networks showed that semantic distance and category switching index increased in the creative condition with respect to the ordinary condition and decreased compared to the random condition. Critically, we showed how children efficiently solved the exploration/exploitation trade-off to generate creative associations by fitting a computational reinforcement learning (RL) model that simulates semantic search strategies. Our findings provide compelling evidence that children balance novelty and appropriateness to generate creative associations by optimally regulating the level of exploration in the semantic search. This corroborates previous findings on the adult population and highlights the crucial contribution of both components to the overall creative process. In conclusion, these results shed light on the connections between theoretical concepts such as bottom-up/top-down modes of thinking in creativity research and the exploration/exploitation trade-off in human RL research.

Significance Statement The ability to generate creative associations between concepts is notoriously increasing with age, reaching its peak during adulthood and decreasing later. However, there is limited evidence in children about the stage of development of these creative problem-solving skills and what are the underlying computational mechanisms. Here, we found 10 years old children are able to solve the tension between novelty and appropriateness when searching for creative associations in a semantic space by optimally regulating the level of exploration in the semantic search. These results corroborate previous findings on the adult population and highlight the relevant contribution of novelty and appropriateness to the overall creative process, shedding light on the connections between theoretical concepts in creativity and human reinforcement learning research.

## Introduction

Children constantly face complex problems in their environment, even though, compared to adults, they have limited knowledge of the world (1) and tend to adopt a more exploratory search strategy when looking for solutions (2–6). The ability to tackle problems by finding an optimal solution has been associated with creative problem solving (CPS) skills (7, 8), which require not exclusively cognitive flexibility but also higher cognitive resources and a broad understanding of facts and constraints (9–11). Indeed, achieving creative solutions rely on the reorganization of existing knowledge, balancing the dual objectives of exploring unknown options and exploiting common ones, as in other cognitive processes (5, 10, 12, 13). Throughout the last decades, researchers have been exploring creative cognition in development and education (14), acknowledging it among the major skills of the 21st century. While CPS is pivotal to human development, its scientific understanding is fundamentally challenging due to its open-ended and internally driven nature. Additionally, whereas many studies have explored the creative process (i.e., CPS) in adults (e.g., 15 to 20), less is known about the developmental processes shaping CPS during childhood. Investigating how children use search strategies when looking for optimal solutions is critically important for developmental and educational scientists, as it can provide us with a crucial understanding of the behavioral principles that lead to creative cognition and healthy development overall.

CPS is semantically imbued (15, 16), involving the search for connections between apparently conflicting or distant concepts and recombining them into new meaningful patterns (15, 16, 17). By definition, creative ideas are distinguished from more routine solutions by their novelty (i.e., originality, unexpectedness), while they are set apart from bizarre and fanciful ideas by their appropriateness, as they must relate to a problem or conform to the requirements of a relevant domain (18, 19). However, the contribution of novelty and appropriateness to CPS is far from being understood (11), especially during developmental age. More than 50 years have been dedicated to studying these processes in children within the field of education (14) albeit empirical results in the literature are inconsistent (20), probably due to measurement and methodological differences across studies.

To overcome this limitation, researchers proposed a reconceptualization of CPS under the computationally informed semantic cognition framework, which relies on two interconnected systems (21, 22): the first is a system of knowledge representation, which enables the storing of semantic concepts; in the second system, stored representations are activated by manipulating them in a goal-oriented and task-dependent manner using semantic control. Notably, such endeavor already has benefited researchers shedding new light on a theoretical and empirical understanding of the CPS (15), especially in terms of semantic memory search strategies (23–28) and toward a robust assessment of CPS potential, refining computational methods to quantify a broad range of verbal tasks (29, 30, 31, 28, 32–34).

A well-established approach to model knowledge representation, inspired by associative theory, is the spreading activation model by Collins and Loftus (35). In this model, knowledge is represented as a network in which concepts are treated as nodes and relations between them are determined by a principle of semantic similarity, the degree to which concepts share essential properties or features. As a result of this prior model, computational models of semantic representation were developed based on the “distributional hypothesis” (36), best captured by Firth (37) sentence “you shall know a word by the company it keeps.” In this model, the distributional properties of concepts are examined in large samples of language data, following the assumption that the more the similarity between two words the greater their occurrence in similar linguistic contexts (statistical redundancy). Concerning differences in knowledge representations over the lifespan, researchers found that children's semantic networks tend to have fewer nodes, connections, clusters, and a greater distance between concepts compared to adults (1, 38, 39). These findings have significant implications for understanding the cognitive processes involved in CPS since how a knowledge system is organized greatly influences information retrieval mechanisms and efficiency (21, 40, 41).

The stored representations in the semantic memory space, on the one hand, can be easily activated. From an early age (42), the passive retrieving of one concept in memory diffusely triggers other connected concepts, from the closest (e.g., “dog” and “cat”) to the most distant (e.g., “dog” and “turtle”) until the patch is exhausted (12, 35). Coherently, studies found that during divergent thinking, many thoughts spontaneously arise (fluency) but manifest highly variable transitions (flexibility) (9, 43, 44), thus facilitating free association through decreased latent inhibition (45). Accordingly, several scholars nowadays have explored individual differences in CPS ability by probing the bottom-up conceptualizations of creative cognition, which heavily rely on associative theories (46, 16) arguing that semantic memory structure mediates CPS performances (15, 47). For instance, recent works found that, throughout the lifespan (48, 49), creative conceptual associations emerge from a flexible (31, 48), small-world semantic network organization which is characterized by highly connected and closer concepts (15, 33, 48, 49); offering a plausible explanation to why creative individuals can easily reach weaker and remote concepts while searching their memory.

On the other hand, within the semantic control system, executive semantic processing has been proposed to aid or constrain the propagation of semantic activation (21, 35). Assuming no differences in associative hierarchy, when creative individuals are prompted for common associations, they are expected to exhibit similar associations while when asked explicitly for uncommon associations, they should show a higher distance between associations due to differences in control strategies (50), as largely demonstrated within adulthood (29, 15, 30, 24, 34). Executive theory of CPS indeed emphasizes a major role of top-down control over attention, ensuring a more efficient memory retrieval through the online monitoring of the search process (e.g. avoiding intrusions and repetitions), leading to the inhibition of ordinary associations and shifting towards meaningful solutions (46, 31, 50). Yet, verbal tasks, such as the verbal fluency or word association tasks (WATs), are frequently used in clinical assessments whose executive functions are impaired (1, 51, 52).

Far from being antagonistic, scholars mainly agree in seeing bottom-up and top-down cognitive processing in a synergistic relationship during the semantic search for creative solutions (46), positing this as a balance between the search for novelty and appropriateness during semantic processing—respectively, alternating between a broad exploratory search process and a process of exploiting nearby concepts to ensure a meaningful solution (8, 12). Likewise, the trade-off between bottom-up/top-down functioning in CPS parallels another trade-off that appears in the reinforcement learning (RL) algorithms literature (exploration/exploitation) (13).

Such literature similarly claims that in many cases, when searching for an optimal solution through a high-dimensional space, the most efficient strategy is to begin with wider exploration and then move to narrower exploitation. Remarkably, researchers suggested that children are more likely to explore the vast space of possibilities when compared to adults (2–6). Unlike adults, children might originate more disparate ideas, switching between ideas less systematically while exploring widely the space of possibilities with a gradual reduction over the lifespan (3–5, 53). Through this behavior, children can pick up information, and gain an understanding of unexpected causal relationships, that adults overlook (2, 3). Even though children's exploratory behavior is well known, their creative outcome is unlikely to be highly valued since the greater variability of the search process might also lead to a higher deviation from meaningful and appropriate solutions (5, 54).

Taken together, previous research highlights differences in children's semantic knowledge size and organization compared to that of adults (1, 38, 39), as well as differences in their search process (2–6, 55), and especially in cognitive control abilities (56, 57); suggesting that children's behavior may differ from that of adults when searching for an optimal solution. What remains to be investigated is how children solve the tension in the context of CPS semantic decisions. Here, we investigate how children navigate the vast space of knowledge representations in order to reach creative associations. To achieve this goal, during a WAT, we varied the demands of cognitive control on associative responses according to CPS criteria. In this way, two control conditions that trigger either a more appropriate (ordinary) or novel (random) response were set, together with the experimental condition that balances the two criteria (creative). We investigated associative responses by modeling them as semantic networks and measured their efficiency (15, 31, 33). Complementary, we quantified the semantic distance between the target word and the associative responses among conditions (30, 34) and extracted a switching index between the selected semantic categories. Ultimately, in order to gain insight into the relationship between bottom-up/top-down and the exploration/exploitation trade-off and create a bridge between creative problem solving and RL scientific community (12), we leveraged the RL framework (58, 59) to model the semantic navigation strategies participants implemented to solve the task.

Hence, consistent with the associative and executive theories of CPS (46, 31, 50) and empirical results on the adult population, we expect that children's creative associations are characterized by more flexibility as compared to the control conditions. Flexibility can thus be considered as a means of both small-world reconfiguration of the creative semantic network (26, 27, 33) and network robustness when undergoing to targeted attack (31, 27, 48). Crucially, by leveraging distributional analysis, we expected the semantic distance of the creative condition to be in the middle of a continuum (29) that has its lowest extreme (low semantic distance) in the ordinary condition and the highest one (high semantic distance) in the random condition (29, 15, 30, 24, 34). In a similar vein, we quantify a category switching index expecting to find the creative condition in the balance between control conditions (29, 43). Finally, by fitting a computational model to estimate the degree of exploration/exploitation for each individual and condition, we expected to find that the creative condition stood in between the ordinary and random condition in terms of the amount of exploration adopted by participants to solve the task.

## Results

**Fig. 1.**
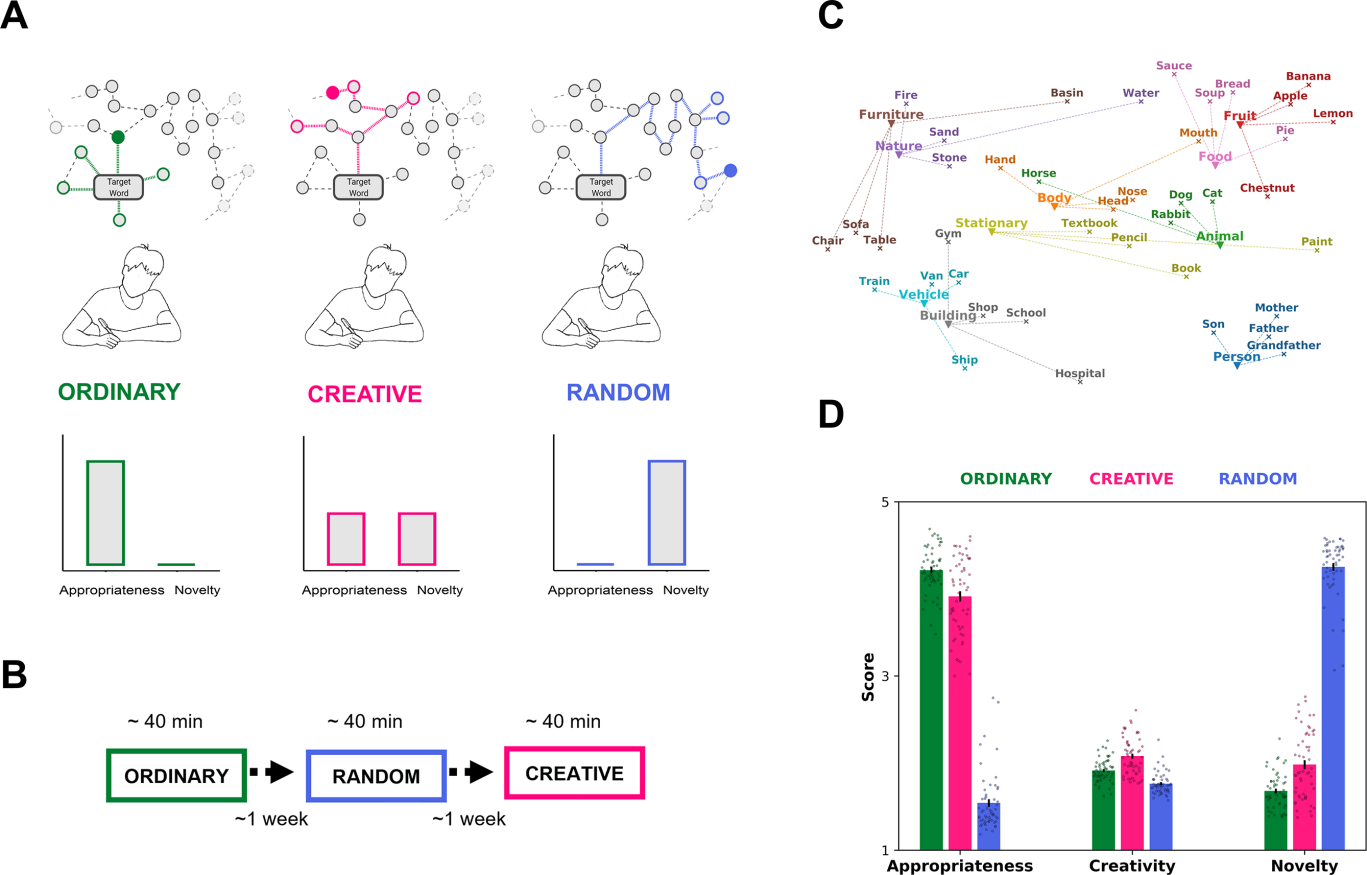
Experimental design, procedure, and selected word stimuli. (a) Participants were instructed to employ different semantic navigation strategies during the WAT, depending on the OR, CR, and RA conditions (top). We represent the possible search processes in a hypothetical knowledge space where the nodes represent the unique concepts, and the edges represent the similarity between these concepts. Nodes with colored borders represent possible answers, those colored inside indicate the answer under consideration. Each condition varied on the inclusion of appropriateness (OR), novelty (RA) search strategies and a balance between these two (CR) (bottom). (b) Participants performed the WAT for approximately 40 minutes, with a 1-week interval between conditions; the order of conditions was fixed while target words were presented in a randomized order. (c) Scatterplot visualizing the selected target words (cross) and their categories (triangles). The 2D coordinates are taken from t-SNE dimensionality-reduced word embeddings (see the "Methods" section). (d) Barplots representing subjective human ratings on appropriateness, creativity, and novelty using a 5-point likert scale. Responses were found to be more creative in the creative condition, which also ranks in the middle range for both novelty and appropriateness with respect to the other conditions. Responses in the ordinary condition were judged more appropriate while those in the random condition more novel. Error bars indicate the SEM.

We collected data from 56 participants (age $M\ = $ 10.05, $SD$ = 0.23) by assessing the WAT. Participants were asked to generate as many related responses as possible to a given target word. We manipulated the nature of these associative responses by asking participants to generate responses according to a set of three instructions, which triggered either a more ordinary (OR), random (RA), or a balanced (CR) response (Fig. 1a and b). By presenting 40 target words per condition, taken equally from 10 semantic categories (Fig. 1c), responses across all the participants and conditions were collected and preprocessed. Because of the open-ended nature of the WAT (i.e., multiple answers were possible for each target word), we checked whether the fluency of responses diverged across conditions by comparing the number of responses generated to the target words between conditions. Across all participants, we collected a total of 21,388 responses, 7734 were generated after the OR condition, 4393 responses after the CR condition, and 9661 after the RA condition. Using a permutation *t* test, we found that participants produced more responses in RA condition ($M\ = $ 172.52, $SD\ = $ 57.36) compared to both the CR ($M\ = $ 78.45, $SD$ = 46.84, Bonferroni-corrected $\mathit{ P}\ = $ <0.001) and the OR conditions ($M\ = $ 130.96, $SD$ = 46.84, $\mathit{ P}\ = $ <0.001). Notably, also the comparison between responses given in OR and CR leads to a significant difference ($\mathit{ P}\ = $ <0.001). Thus, we controlled for this confounding fluency factor in the subsequent analyses appropriately. We also collected human ratings for testing whether the responses in the creative condition were generated by following a balanced strategy between novelty and appropriateness (Fig. 1d). Here, we asked eight raters to indicate a score on three scales, namely novelty, appropriateness, and creativity. There was a substantial agreement among the raters, measured by the intra class correlation coefficient (ICC) with the two-way fixed-effects model ( $IC{C}_{novelty} = \ 0.93,\ \ IC{C}_{appropriateness} = \ 0.96,\ \ IC{C}_{creativity} = \ 0.61$). Thus, we tested whether participants’ responses in each condition were judged by the raters as aligning to the instruction set. Indeed, we found that the appropriateness was higher in the OR condition compared to both CR and RA conditions (all $\mathit{ P}$ <0.001). We also found that novelty was higher in the RA condition with respect to CR and OR (all $\mathit{ P}$ <0.001) and that the creativity score was higher in CR compared to OR and RA conditions (all $\mathit{ P}$ <0.001, for all statistics see Table S2).

## Semantic Networks Organization

**Fig. 2.**
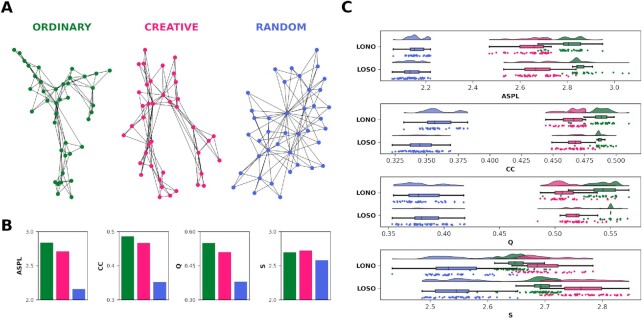
Topological quantifiers of the semantic networks. (a) A 2D visualization of the undirected, unweighted semantic networks of the OR, CR, and RA conditions. The nodes represent the target WAT words, and the edges represent the cosine similarity between unique responses given by participants. (b) Barplots depicting the topological quantifiers of the full networks. (c) Raincloud plots represent the results from the leave-one-node-out (LONO) and leave-one-subject-out (LOSO) procedures on the topological quantifiers. All *P* < 0.001 (Bonferroni corrected).

Data were first analyzed using a network science-based approach (33, 60), which enables us to explore the organization of the conceptual network depending on how often the responses co-occur across the sample in each condition. We constructed a group-level semantic network for each condition structuring the data into a matrix in which columns represent the target words, rows indicate the preprocessed unique association responses, and cells contain the amount of participants generating a response to a target word. Then, we computed the cosine similarity between the vectors associated with each target word in a pairwise fashion. The higher the number of similar associations generated by participants between pairs of target words and the greater the number of participants generating these associations, the stronger the association between them (link weight). This procedure resulted in an undirected weighted semantic network for each condition, with the target words as nodes and the cosine similarity as links. After filtering the networks (61), their organization was analyzed using the following network quantifiers: clustering coefficient (CC) (62, 63), average shortest path length (ASPL) (62), modularity index (Q) (64), and small-worldness measure (S) (62). Results from the networks’ comparisons revealed qualitative (Fig. 2a) and quantitative (Fig. 2b and c) differences in their structures between conditions. The semantic graph of the CR condition showed lower level of organization (ASPL = 2.71 and Q = 0.51) and higher flexibility (S = 2.72) compared to the network of the OR condition (ASPL = 2.84, Q = 0.55, and S = 2.70). Similarly, RA showed a lower level of organization (ASPL = 2.16 and Q = 0.38) as well as a lower S value (2.58) compared to the CR condition. The CC measure was higher in the OR network (0.49) than in both the CR (0.47) and RA (0.35) networks.

We statistically examined the validity of our findings by applying two complementary approaches, the LONO and LOSO. In the LONO procedure, we iteratively computed the topological quantifiers on the partial networks resulting from the exclusion of one node at each iteration, for each node. The LOSO procedure iteratively excludes one participant and then repeats the entire process of building semantic networks for each subject. Thus, we computed the topological quantifiers on the resulting partial networks. A permutation *t* test was computed on each measure for comparing the conditions in both the LONO and LOSO procedures. Results indicated that CR had a significantly lower ASPL, CC, Q, and higher S compared to OR, whereas significantly higher ASPL, CC, Q, and S were found in CR in comparison to the RA condition (all $\mathit{ P}$ <0.0001). Overall, these findings confirm the analyses on the full networks, all with very large effect sizes measured by Cohen's *d* (Fig. 2c; for statistics see Table 1).

**Table 1.** Semantic networks topology quantifiers and results from the paired two-tailed permutation *t* test of the partial networks comparing the CR, OR, and RA conditions.

	OR	CR	RA	CR vs. OR		OR vs. RA		CR vs. RA	
Meas	$M( {SD} )$	$M( {SD} )$	$M( {SD} )$	*t*	*d [ci 95%]*	*t*	*d [ci 95%]*	*t*	*d [ci 95%]*
(a) Full network									
ASPL	2.84	2.71	2.16						
CC	0.49	0.47	0.35						
Q	0.55	0.51	0.38						
S	2.70	2.72	2.58						
(b) LONO									
ASPL	2.81 (0.06)	2.64 (0.07)	2.16 (0.04)	13.893	2.52 [1.93, 3.11]	55.363	13.15 [11.06, 15.23]	33.717	8.31 [6.95, 9.67]
CC	0.49 (0.01)	0.47 (0.01)	0.36 (0.01)	14.896	2.39 [1.81, 2.96]	51.022	11.86 [9.97, 13.75]	39.943	9.93 [8.33, 11.53]
Q	0.54 (0.02)	0.51 (0.02)	0.38 (0.02)	10.937	1.91 [1.38, 2.43]	41.274	9.92 [8.32, 11.51]	37.026	7.58 [6.33, 8.83]
S	2.65 (0.03)	2.70 (0.04)	2.55 (0.05)	6.681	1.49 [0.99, 1.98]	12.187	2.57 [1.98, 3.16]	17.137	3.41 [2.72, 4.10]
(c) LOSO									
ASPL	2.86 (0.05)	2.67 (0.08)	2.14 (0.04)	14.689	2.93 [2.40, 3.47]	84.943	15.79 [13.69, 17.89]	47.009	8.85 [7.63, 10.06]
CC	0.49 (0.01)	0.47 (0.01)	0.35 (0.01)	14.017	2.80 [2.28, 3.32]	91.422	16.49 [14.30, 18.69]	62.365	11.96 [10.35, 13.57]
Q	0.55 (0.01)	0.52 (0.01)	0.38 (0.02)	17.440	3.30 [2.73, 3.87]	74.016	13.06 [11.31, 14.81]	54.599	9.31 [8.04, 10.59]
S	2.69 (0.03)	2.76 (0.04)	2.55 (0.04)	10.405	2.0 [1.55, 2.45]	22.250	4.00 [3.35, 4.64]	27.443	5.33 [4.54, 6.13]

ASPL; CC; Q; and S. LONO; LOSO. The t-statistics (from a parametric *t* test student) and Cohen's ${\boldsymbol{d}}$ (65) values are presented. Cohen's d effect sizes: 0.20, small; 0.50, moderate; 0.80, large; 1.10, very large. All *P* < 0.0001, corrected for multiple comparisons using Bonferroni (0.017).

To get rid of possible associative fluency confounds on network structure, we also repeated the above network analysis considering only the first two associative responses given by all participants to a target word (33). Results from this subset are in line with the results relying on the full dataset (see Table S3 in Supplementary Materials for statistics), corroborating the validity of the findings.

## Networks Percolation

**Fig. 3.**
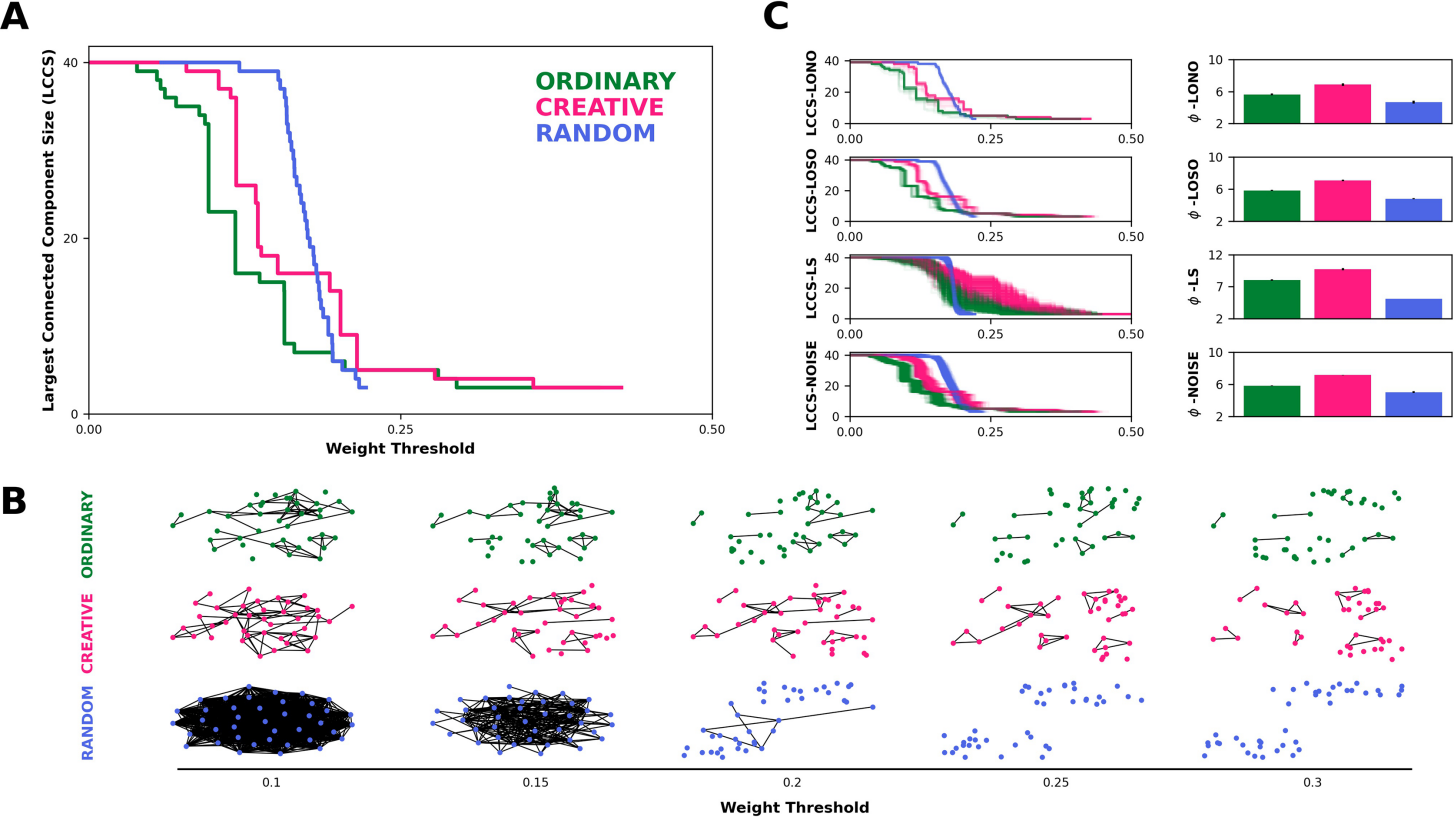
Network percolation. (a) Line plot representing the percolation process of the CR (pink), OR (green), and RA (blue) full networks. The x-axis represents the weight threshold, starting from the smallest weight in a network (0.01) to a weight strength in which the giant component is smaller than three nodes (0.5). (b) OR, CR, and RA semantic networks undergoing the percolation process, visualized at different weight thresholds. (c) On the left, line plots of the LONO, LOSO, link shuffling (LS), and link noise procedures. Each line is an iteration, colors encode the conditions. On the right, barplots show the percolation integral $\phi $ between conditions and across the three procedures. Error bars represent the SEM.

Next, we performed a percolation analysis (31) to investigate the flexibility of the semantic networks, probing the network's resilience under targeted attacks. Here, we assumed that the higher the network robustness is, the higher its cognitive flexibility (31). Thus, we used the weighted networks previously constructed from the preprocessed data (we controlled for possible associative bias in response fluency (31), see the "Method" section). To avoid possible confounders and retain more information, we used in this analysis the unfiltered similarity matrices. Throughout the percolation analysis, networks are “attacked” by removing all the edges whose weight strength falls below a threshold, called the percolation step. Each time a percolation step occurs, we measure the size of the largest connected component (LCCS), namely the largest cluster of nodes connected only to each other. Once the percolation procedure was completed, we computed the percolation integral ($\phi $), which is the area under the curve representing the LCCS across the percolation steps. Our analysis revealed that the CR network was more robust to network percolation, as exhibited by a higher percolation integral ($\phi $ = 7.09), with respect to both OR ($\phi $ = 5.84) and RA ($\phi $ = 4.83), as shown in Fig. 3a. We illustrated in Fig. 3b the network appearance throughout the percolation process, focusing on how the LCCS differed across conditions at various percolation steps, and indicating that the OR and RA networks broke apart faster than the CR networks.

To determine the statistical significance of our findings, we implemented four approaches: LOSO, LONO, LS, and noise analyses (NA). As mentioned above, when computing the LONO and LOSO procedures, we iteratively excluded each node or participant and ran the percolation process on the resulted networks computing $\phi $. In order to control for the possibility that differences between networks may stem from the differences in the link weights, the LS analysis was conducted by randomly exchanging pairs of links in the network, running the percolation process, and computing $\phi $. Lastly, NA was computed by adding gaussian noise to the link weights and then analyzing the $\phi $. Overall, these analyses indicated consistent results, meaning that the average percolation integral $\phi {\rm{\ }}$of the CR network was significantly larger than the OR and RA networks (all $\mathit{ p}$ <0.0001) with effect sizes ranging from moderate to very large (see Fig. 3c and Table 2 for statistics).

**Table 2.** Results from the two-tailed paired-sample permutation t-test on percolation integral comparing conditions.

	OR	CR	RA	CR vs. OR		OR vs. RA		CR vs. RA	
	$M( {SD} )$	$M( {SD} )$	$M( {SD} )$	*t*	*d [ci 95%]*	*t*	*d*	*t*	*d [ci 95%]*
Full Net	5.84	7.09	4.83	-					
LONO	5.64 (0.11)	6.86 (0.14)	4.67 (0.18)	64.013	9.59 [8.04, 11.14]	28.114	6.61 [5.50, 7.73]	57.409	13.57 [11.42,15.72]
LOSO	5.83 (0.07)	7.09 (0.1)	4.8 (0.08)	82.366	14.70 [12.74, 16.66]	77.617	12.96 [11.22, 14.69]	149.436	25.54 [22.17,28.90]
LS	8.02 (0.38)	9.72 (0.51)	5.1 (0.05)	58.852	3.77 [3.56, 3.97]	168.558	10.77 [10.28, 11.26]	202.997	12.83 [12.25, 13.41]
Noise	5.81 (0.08)	7.16 (0.1)	5.02 (0.3)	230.080	14.80 [14.14, 15.46]	55.247	3.57 [3.37, 3.77]	152.239	9.53 [9.09, 9.96]

Results from the paired two-tailed permutation*t* test on percolation integral comparing the CR, OR, and RA conditions. LONO; LOSO. FullNet . L; T-statistic estimated from a parametric *t* test. Cohen's ${\boldsymbol{d}}$ effect sizes: 0.20, small; 0.50, moderate; 0.80, large; 1.10, and very large. All P < 0.0001 corrected for multiple comparison using Bonferroni (0.017).

## Distributional Semantics

**Fig. 4.**
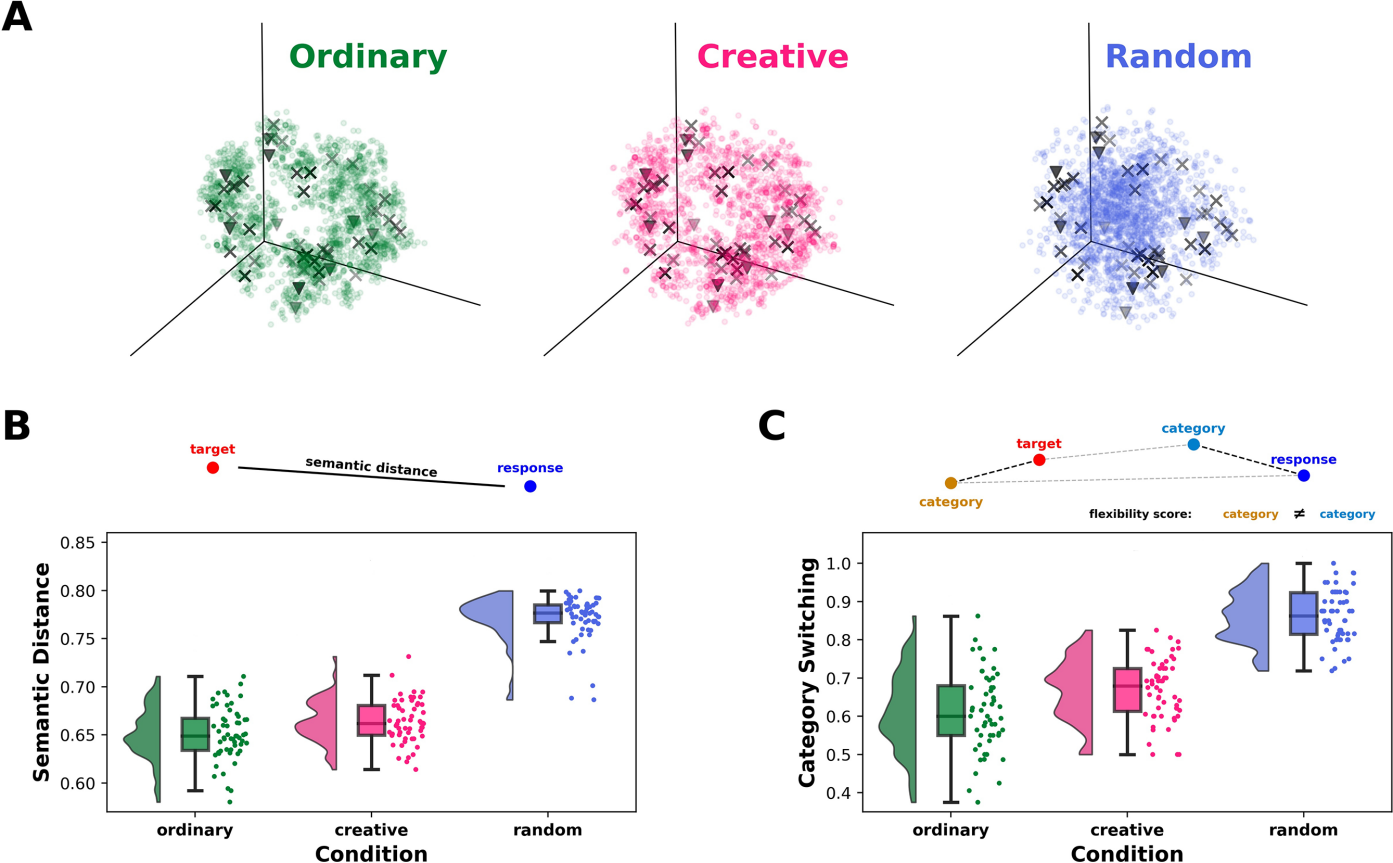
Semantic distance and category switching. (a) t-SNE scatter plots representing the preprocessed data among the conditions in the embedding space. Responses are coded as dots, target words as cross and category words as triangles. (b) Raincloud plots representing the semantic distance between conditions. (c) Raincloud plots representing the category switching between conditions.

Then, we also analyzed the data using a distributional semantics approach. According to this model, semantically similar words tend to occur in similar contexts (36). For methodological robustness, in this study, we used three pretrained word2vec models (66) trained with different methods (CBOW or Skip-gram) and different Italian text corpora. To ensure a reduced influence of specific models, while increasing the reliability of the results (67), we averaged the embeddings of the target words, category, and participant responses across all models. In Fig. 4a, we depicted the associative responses’ word embeddings of each condition in a 3D space using the t-distributed stochastic neighbor embedding (t-SNE) algorithm. We found that, qualitatively, CR responses tend to be more clustered around target words with respect to RA but less clustered compared to the OR condition. To quantify this intuition, for each target-response pair of word embedding vectors, we computed the semantic similarity as the cosine similarity, subtracted it from 1 to obtain a semantic distance score. Then for each participant, we averaged the semantic distances of all target words for each condition. Permutation *t* tests were computed on the resulted semantic distance score to compare the conditions. Results indicated that the semantic distance in the CR condition ($M\ = $ 0.66, $SD\ = $ 0.02) was significantly lower than the RA condition ($M\ = $ 0.77, $SD\ = $ 0.02, Bonferroni-corrected $\mathit{ P }< $ 0.0001, $d\ = $ 4.61, 95% CI [3.9, 5.32]) and significantly higher compared to the OR condition ($M\ = $ 0.65, $SD\ = $ 0.03, $p\ = {\rm{\ }}$0.003, $d\ = {\rm{\ }}$0.49, 95% CI [0.12, 0.87]) (as illustrated in Fig. 4b and reported in Table 3 and 4). Additionally, we calculated a category switching index by computing the semantic distance between a target word and all the selected category words as well as between the response of that target and the categories. We assign a category to the target and the response words as the one that has the minimum semantic distance. For statistical comparison, we averaged category switching values across targets for each participant and condition. Permutation *t* tests were computed on category switching scores to compare the conditions. As a result of this analysis (Fig. 4c and Table 3), during the CR condition, the category switching score ($M\ = $ 0.68, $SD\ = $ 0.07) was significantly higher compared to the OR condition ($M\ = $ 0.65, $SD\ = $ 0.08), whereas both CR and OR showed significantly lower category switching scores in comparison with the RA condition ($M\ = $ 0.88, $SD\ = $ 0.05). Statistically significant differences in category switching scores between conditions were observed among all comparisons, with effect sizes measured by Cohen's *d*, ranging from small when comparing CR vs. OR for category switching (*p* = 0.013, *d* = 0.39, 95% CI [0.02, 0.77]), to very high when comparing CR vs. RA and OR vs. RA (see Table 4 for statistics). With the aim to control for possible associative fluency confounds on semantic fluency and category switching indexes, we also repeated the above pipelines considering only the first two responses of the WAT. In line with full dataset findings, results showed significant differences for both semantic distance and category switching, placing the CR condition values in between the OR (lower score) and RA (higher score) conditions (see Tables S4 and S5 in Supplementary Materials for statistics).

**Table 3.** Descriptive statistics for semantic distance and category switching.

	OR	CR	RA
	$M( {SD} )$	$M( {SD} )$	$M( {SD} )$
Semantic distance (SD)	0.65 (0.03)	0.66 (0.02)	0.77 (0.02)
Category switching (CS)	0.65 (0.08)	0.68 (0.07)	0.88 (0.05)

**Table 4.** Results from comparisons across conditions using the two-tailed paired-sample permutation *t* test on semantic distance and category switching.

	CR vs. OR			OR vs. RA			CR vs. RA		
	*t*	*p*	*d [ci 95%]*	*t*	*p*	*d [ci 95%]*	*t*	*p*	*d [ci 95%]*
SD	2.98	0.003	0.49 [0.12, 0.87]	21.762	<0.0001	4.63 [3.92,5.34]	22.714	<0.0001	4.61 [3.9, 5.32]
*CS*	2.508	0.013	0.39 [0.02, 0.77]	16.94	<0.0001	3.59 [2.99,4.19]	16.462	<0.0001	3.36 [2.79, 3.94]

Results from the paired two-tailed permutation *t* test on SD and CS comparing the CR, OR, and RA conditions. T-statistic estimated from a parametric *t* test. Cohen's ${\boldsymbol{d}}$ effect sizes: 0.20, small; 0.50, moderate; 0.80, large; >1.10,and very large. All *P* values corrected for three paired comparison using Bonferroni (0.017).

## Computational Modeling

**Fig. 5.**
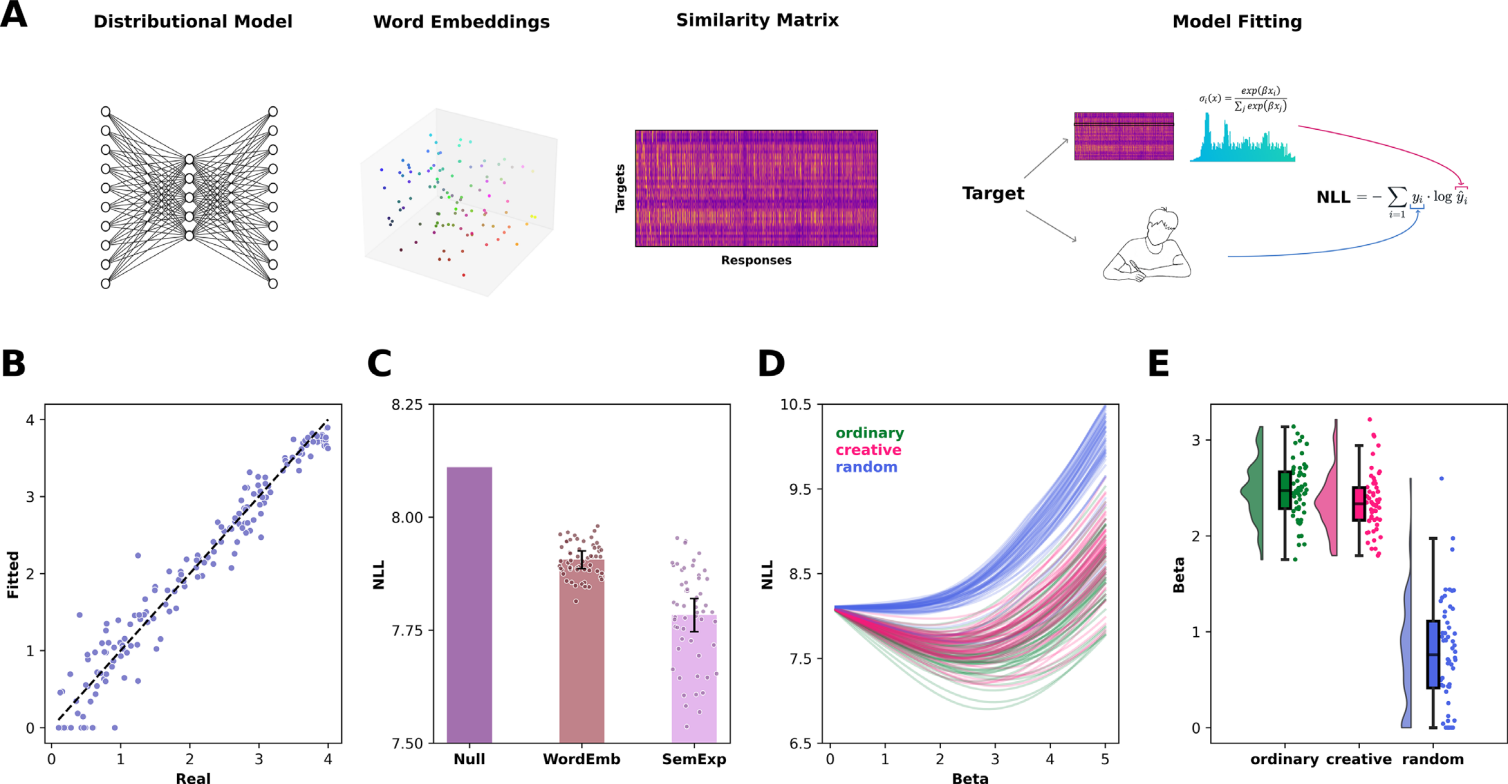
Computational modeling. (a) Description of the computational modeling analysis pipeline. From left, we took word2vec distributional semantics models and get from them word embeddings vectors of target and unique responses words. Then, we computed a similarity matrix having the target words as rows and responses as columns. We used this matrix, together with an action selection rule (softmax function), to build the semantic explorer model and fit it to the children data using the NLL loss function. (b) Scatterplot of the parameter recovery analysis, showing on the x and y axis the real and fitted parameters, respectively. (c) Barplots showing the loss values of the three models. The null model does not have variability since it has a fixed uniform distribution over the response selection. Errorbars indicate SEM. (d) Lineplots depicting the loss landscape, computed using a grid search procedure as a function of the beta parameter. Each line represents a single participant on each condition. (e) Raincloud plots representing the beta parameter between conditions.

Finally, we leveraged the RL framework (58, 59) to analyze the semantic navigation strategies implemented by children to generate an associative response to a given target word. Therefore, we propose a computational model that is able to capture the amount of exploration that children adopted in a semantic space (Fig. 5a). The computational model, which we called the Semantic Explorer model (SemExp), consists of two components. The first one is a similarity matrix between target words and all the unique responses given by the whole sample. This matrix is created from the averaged word embeddings previously used for the distributional semantic analysis (Fig. 5a) and can be seen as an approximation of a value function operating in a semantic state space (59). The second component is an action selection rule that generate the response word given a target word. This selection is performed by using the softmax function on the vector of similarity values of a given target word. To control the amount of exploration versus exploitation, there is an inverse temperature parameter, which we called beta (*β*), that determines the degree to which the value function influences choice. In other words, it controls the level of stochasticity in the response generation process, where lower values of *β* induce the model to select responses more randomly (i.e., *β* <1 means more exploratory behavior) and a higher β allows the model to deterministically generate the highest value response word (i.e,. *β*>1 means more exploitative behavior). Model fitting was performed using the negative log-likelihood (NLL) as loss function and gradient-based methods for the parameter estimation procedure (Fig. 5a, see methods for further information). Before fitting the SemExp model to the participants’ choices, we checked whether the amount of data collected was enough for reliably estimating the *β* parameter by performing a parameter recovery analysis (Fig. 5b). We found that the correlation of real and fitted *β* parameters was very high (*r* = 0.97 *p* < ${10}^{ - 109}$). Next, we performed model selection by comparing three models (Fig. 5c), a null model with always a uniform distribution for generating responses, a fixed model (WordEmb) relying only on the first component of the SemExp model (the similarity matrix built on the word embeddings shown in Fig. 1a) and the SemExp model with the free parameter β fitted for each participant and condition. Results showed how the SemExp model significantly performed better at explaining children's response data compared to both the null model (*p* < 0.0001, *d* = 5.44, 95% CI [4.63, 6.24]) and the WordEmb model (*p* < 0.0001, *d* = 2.33, 95% CI [1.85, 2.81]). Notably, the WordEmb model significantly outperformed the null model (*p* < 0.0001, *d* = 7.65, 95% CI [6.58, 8.72]), providing evidence that our methodological choice of averaging the word2vec models was reliable (Fig. 5c). After confirming that SemExp was the best model among candidates, we visually inspected the loss landscape as a function of the beta parameter using a grid search procedure (Fig. 5d). We observed how the RA condition substantially diverged from the other two, confirming the previous results on the distributional semantics analysis. Finally, we extracted the *β* parameters for each participant and condition and tested their differences. We found that the beta in the CR condition (mean ${\beta }_{CR} = \ 2.34$) was significantly lower compared to OR (mean ${\beta }_{OR} = \ 2.47$, *p* = 0.0058, *d* = 0.41, 95% CI [0.04, 0.79]) and significantly higher compared to RA (mean ${\beta }_{RA} = \ 0.76$, *p* < 0.0001, *d* = 3.44, 95% CI [2.86, 4.03]). We also observed OR to be significantly higher compared to RA (*p* < 0.0001, *d* = 3.73, 95% CI [3.11, 4.34]).

## Discussion

In the present study, we investigated efficient information search during childhood from the semantic cognition perspective. To achieve this goal, we used a within-subject design, administering a WAT while varying the demands of cognitive control on associative responses. Our paradigm involves three conditions that differ for the inclusion of the CPS’ defining criteria (29): i.e., appropriateness (OR condition), novelty (RA condition), and a balance between these two (CR condition). Our task provided us to address several related questions.

We first adopted a network science approach to explore the nature of associative responses by modeling them as conceptual networks for each condition. Within this framework, we measured and compared the networks’ efficiency in terms of both structural properties and resilience when undergoing percolation analysis (15, 31, 33). We found that children's associative performance in the creative condition significantly leads to increased small-worldness, compared to both the OR and RA conditions. Our results are consistent with previous studies demonstrating that high levels of small-worldness mark a maximization of network efficiency (68), associated with higher levels of CPS and cognitive flexibility, in both adults (69, 33, 48) and children (49). More in-depth evidence of the efficient structure of the CR network is provided by the comparison of all remaining topological quantifiers, whose values consistently lie in between the OR and RA networks.

At one end of this continuum, the OR network shows the most rigid organization (51), characterized by a better local organization of associations but also by less-interconnected nodes and greater modularity (i.e,. highest CC, ASPL, and modularity, respectively). This is in line with our expectation since the network configuration during the OR condition was guided by the inclusion of only the appropriateness criteria, which increased the likelihood of activating nearby nodes while constraining the search and inhibiting the spreading of activation. The rigidity of such network configuration has been well-documented in the literature (41). For example, some studies found this pattern to be advantageous for information search, predominantly encountered in individuals with high deductive reasoning scores both in children (49) and adults (69, 70, 71). More often, networks with higher ASPL and Q tend to reflect an impairment in the efficiency of the search process (1, 51). For instance, individuals with high-functioning autism have been found to have a more modular semantic network than neurotypical controls (51). A possible explanation proposed by the authors is that higher levels of segregation could make it more difficult to integrate information since the network breaks apart into smaller subparts.

When compared to OR, the CR network showed lower ASPL and modularity, which in turn has been linked with higher network flexibility. The CR semantic network configuration has already been linked to both higher levels of CPS abilities (15, 33) and cognitive flexibility (27, 48). As compared to CR, the RA network significantly decreased in all topological quantifiers.

Indeed, at the other end of the continuum, the RA network was the most chaotic, having locally poorly connected nodes, a shorter average distance between nodes, and the lowest modularity (i.e., lowest CC, ASPL, and modularity, respectively). Although a chaotic semantic network state allows for more flexible creative processing, here we interpret the findings on the RA network as representative of an over-flexible semantic memory state (72). Chaotic networks have been described as random networks, i.e., networks that are highly connected but poorly organized (72), assuming that all connections between two nodes are random and uniform (73). A similar network topology and language disorganization is a distinctive feature in psychosis (52).

Consistently, the percolation analysis revealed that the CR semantic network was significantly more resilient to the percolation process as compared to both control conditions. When undergoing a targeted attack, the CR network breaks apart more slowly than the OR and, in turn, the OR showed more robustness than RA, as indicated by a larger percolation integral. Again, these results corroborate those already found in the literature, linking this behavior in semantic networks with cognitive flexibility and CPS (31, 27, 48).

Another goal of this study was to clarify the role of semantic distance in light of the novelty-appropriateness trade-off during childhood. Since CPS relies on the notion that farther associations are more creative (16), we leveraged the distributional semantics approach using different pretrained word2vec models, to probe how semantic distance can disentangle the different search strategies adopted between conditions. Additionally, with the aim to quantify the number of deviations in categories tapped by responses, we proposed a new approach to quantify category switching based on distributional models.

Here, results from the distributional analysis showed that CR responses increased in both semantic distance and category switching rate with respect to OR responses. These findings acknowledge results from other studies regarding the role played by divergent thinking in achieving farther associations and reaching diverse categories among adults (30, 43, 44). However, when compared to RA, the CR associations were less distant, also showing less category switching. This is due to the more exploratory nature of the RA condition, which accounts solely for the search for novelty in the association. Critically, in contrast to the conventional assumption that novelty characterizes CPS, we showed the crucial prerequisite of the appropriateness criterion in understanding CPS, as a few previous studies have already highlighted (11, 29).

Finally, we adopted a computational modeling approach to gain insights on the computational mechanisms operated by children for navigating the semantic space and generate responses given the target words and instruction set. We leveraged the RL framework (58, 59) and proposed a semantic explorer model able to generate linguistic responses for the WAT. To the best of our knowledge, our model is the first in the literature aiming to provide a computational generative model for this task, exploiting state-of-the-art computational frameworks such as deep learning (66), for building the first component (semantic similarity matrix) of the SemExp model, and RL (59), for implementing the second component (action selection rule). Critically, our results provided insights on the semantic navigation strategies adopted by children, since in the RA condition the observed mean sample value of*β* was 0.76. This indicates that children's response generation policy was indeed dominated by an exploration factor, given that a value of *β* < 1 is commonly considered as indicating exploratory behavior (59). Crucially, we observed that in the CR condition the observed mean sample value of *β* was 2.34, indicating that children were exploiting their semantic knowledge to generate creative associations, but were more exploratory when compared to the OR condition (mean *β* = 2.47). Importantly, these findings help to create a bridge between different communities we believe being tightly connected, namely the creative problem-solving and the RL communities. We hope these results will shed more light on the connections between theoretical concepts such as bottom-up/top-down modes of thinking in creativity research and the exploration/exploitation trade-off in human RL research.

In summary, our study provides evidence for different kinds of results supporting optimal memory search during developmental age. The associations in the CR condition led to a more flexible and robust semantic network, as well as a balanced semantic distance and category switching compared to control conditions. Notably, in the CR condition, children's performance was closer to the OR than to RA (in terms of both networks and distributional semantics measures). This pattern is opposite to what Heinen and colleagues (29) found in the adult population, where the semantic distance in their creative condition was closer to the random condition. A possible explanation for this could be the fact that in children of the age of our sample, the vocabulary size is poorer than in adults, therefore it is generally more difficult for them to move away very far in the semantic space from the target word. In addition, viewed in the light of the recent developmental literature based on optimality search, newfound research also demonstrated that, even at the youngest age range, children do not solely rely on random exploration instead they are able to efficiently adapt their search behavior (5, 74–76).

Several limitations need to be taken into consideration. The first relied on observed differences in the fluency of word association, probably due to either difference in cognitive load between conditions, or individual differences in verbal fluency. Despite we dealt with this issue throughout the analyses, further studies are encouraged to control for this factor, for instance using a fixed range of responses (29). Also, a possible confounding factor could be the order of presentation of the conditions. Although we followed the logic of previous studies, highlighting the importance of not presenting first the creative condition, we encourage future studies to investigate the importance of counterbalancing the conditions’ order. Next, as already pointed out in the literature (30), when cued to be random, participants’ word associations may be artificially modulated by using different strategies (e.g., finding inspiration around the room, following category-based or phonological-based strategies), affecting the final score. A possible intervention in this regard could be to check the used strategy by asking participants at the end of the task. Another limitation is that we do not directly compare our data to those of adults, nor between different ages during development. Finally, a possible improvement could be to increase the number of measures used. Given the parallels with other trade-offs, such as the exploration-exploitation, deliberate/controlled cognitive processes, or more broadly, the crucial role of executive functions in CPS, future studies could replicate and extend our results by implementing other measures and tasks.

In conclusion, our findings provide evidence that 10-years-old children implement CPS similarly to adults, efficiently solving the tension between a conventional and a chaotic semantic search strategy. Furthermore, we corroborated the notion that CPS can be fully investigated only when both novelty and appropriateness components are considered. Overall, our findings lay a bridge between developmental science, and the creative and semantic cognition communities, promoting a multidisciplinary approach to the investigation of how CPS skills develop in humans and providing a critical understanding of the behavioral principles underlying creative cognition during childhood.

## Materials and methods

### Participants

We recruited 61 participants from a primary school in Rovereto (Italy). Children were thoroughly screened to exclude individuals with a history of neurological diseases and learning disabilities affecting language. Two volunteers were excluded from the analysis because gave incomplete fulfillment of the data collections. The final pool results of 56 healthy children (32 male), Italian language speakers, aged between 10 and 11 years ($M\ = $ 10.05, $SD$ = 0.23). No statistical methods were used to predetermine sample sizes, but our sample size is comparatively similar to experimental studies adopting a within-subject design (77). A parent or legal guardian provided informed consent for their children's participation. The study protocol was approved by the Human Research Ethics Committee of the University of Trento (prot. 2018–027).

### Procedure

The experiment consisted of three conditions where participants performed the WAT following different instructions (see (29) for similar procedures). Following previous studies (29, 78), conditions were always administered in the following order: OR, RA, and CR. The target words (*n* = 40), within each condition, were randomized across participants. The WAT was administered as a paper and pencil task. The study was conducted in the classroom, during usual class hours, from 2019 January to March (completed in groups of ∼20 participants). During the experiment, volunteers were spaced apart and supervised by two of the authors. Each condition lasted 1 hour and was collected on different days (∼1-week interval between conditions); with a total duration of ∼3 hours.

### WAT

In the WAT, participants had one minute to generate as many related responses as possible to a given target word. Participants were asked to respond using a single or a compound word and avoiding proper names. In this task, 40 target words were presented in each condition (Fig. 1c), belonging to 10 categories, and counterbalanced for the domain in natural and artifact concepts (79, 80). Semantic categories were selected a priori from a list of 32 Italian production norms gathered by (81) and accordingly to the most commonly used categories in the cognitive neuroscience literature (80, 82, 83). Target words were extracted from the Lexvar database (84) and controlled for concreteness, familiarity, and imageability. See the "Supplementary Materials" section for a detailed description of the stimuli selection procedure (Table S1). Before analyzing the data, we preprocessed the raw responses to the WAT by excluding idiosyncratic answers and nonwords and controlling for other possible confounds (i.e., spell-checked, converted plural words into singular, transformed to lowercase, cleared of nonalphabetic tokens and stop-words). Additionally, responses that contain the target words were not included in the analysis.

### Human rating analysis

We collected human ratings from eight raters on a subsample of our data. We presented the unique responses of 10 out of 40 target words (one for each category) to each rater and ask them to rate with a 5-point Likert scale (ranging from not at all as 1 to very much as 5) each target-response pair on three scales, namely novelty, appropriateness and creativity. To estimate the inter-raters reliability, we computed the ICC among all the raters on each scale across all the target-response pairs. Then, we averaged the raters’ scores on each scale for each participant and condition. We used a two-tailed paired-samples permutation *t* test analysis ($\alpha $ = 0.05 adjusted using Bonferroni correction for three pairwise comparisons $\alpha $ = 0.017; 10,000 iterations) to investigate the statistical differences between conditions for each scale.

### Semantic network structure analysis

First, we modeled the WAT responses as a group-based network using a recently developed (60) yet applied framework (15, 33, 48, 49). In each network, nodes represent target words, and edges represent relations between two vertices. This link is based on the extent to which participants generated similar associative responses to a pair of target words and the number of participants who generated similar associative responses (33). Accordingly, the greater the number of similar associations generated between a pair of target words and the larger the number of participants who generated these associations, the higher the strength between the pair of words. In order to construct the semantic network, we structured the data into a *n*×*m* matrix for each condition, in which each column *m* represents the target word (i.e., nodes), and each row *n* represents a unique response given by the entire sample. On each cell, responses were encoded using the number of participants which provided the response *n* to the target word *m* and 0 otherwise.

Thus, we constructed a word-similarity matrix by computing the cosine similarity between the vectors associated with each target word in a pairwise fashion. We obtained an adjacency matrix of a fully connected, weighted network, with target words as columns and rows, and cells as the weights of the links between the words. To ensure that only relevant information is retained in the network, we removed spurious associations (i.e., weak similarity) by filtering the adjacency matrices with the Triangulated Maximally Filtered Graph (TMFG) method (61). The result was an undirected weighted semantic network containing the target words as nodes and the cosine similarity as links, for each condition.

We performed thorough descriptive analyses of the structure of the conditions’ semantic networks. We calculated the following topological measures on each network: the CC (63), ASPL(85), Q (64), and S (62). The CC measures how closely the nodes in a network cluster together, and it is considered a measure of connectivity. The higher the CC, the better the local organization, and the stronger the connection within the network. Here, network clustering is based on transitivity (63). The ASPL determines the average number of steps required for all possible pairs of network nodes. A lower ASPL value may help reach relatively remote nodes faster (85). The Q evaluates how a network is split into subnetworks (64). Finally, we computed the small-worldness of the conditions’ semantic networks to provide additional context to the CC and ASPL results. Indeed, since small-world networks are highly clustered (e.g., lattice graphs), with an averaged short path lengths (e.g., random networks), a network *G* with *n* nodes and *m* edges is defined as a small-world network (85) if it has a similar path length but greater clustering of nodes than an equivalent Erdös-Rényi random graph with the same *m* and *n* (62). In this way, the S indicates the extent to which nodes that are not directly connected can be reached through connections between their neighbors.

Two complementary methods of statistical analysis were used, LONO and LOSO. Implementing the LONO approach, we constructed several partial networks resulting from the exclusion of one node at each iteration, for every node (*n* = 40). Then we calculated the before mentioned network measures on the partial networks. In a similar vein, applying the LOSO procedure, we excluded one participant at each iteration and repeat the entire pipeline for building the semantic networks and computing the network measures, for every participant (*n* = 56). We used a two-tailed paired-samples permutation *t* test analysis ($\alpha $ = 0.05 adjusted using Bonferroni correction for three pairwise comparisons $\alpha $ = 0.017; 10,000 iterations) to investigate the statistical differences between conditions for each measure in both LONO and LOSO. We also used Cohen's *d* as a measure of effect size. Network analyses were conducted in R and Python using the NetworkToolbox package (86) and the NetworkX library (87), respectively; inferential statistics and data visualization were implemented in Python.

Additionally, to control for associative fluency bias, we performed the above network analysis only based on the first two associative responses given by each participant to a target word (33). We first selected a subset of the raw response's dataset, including the first two responses given by a participant to each target word and for each condition. Afterward, we analyzed differences between conditions in semantic networks only considering the first two association responses from the sample.

### Network percolation analysis

Network percolation analysis enables us to estimate networks’ robustness and their structure flexibility (88), inferred by how fast the network breaks apart under a targeted “attack” (31). In the percolation analysis, networks are “attacked” by removing edges that fall beneath a certain threshold, named the percolation step (31). Here, the smallest weight in the network was used as the initial threshold, and the threshold resolution was computed as the lowest difference between the sorted weights. At each percolation step, we measure the LCCS, which is the size of the LCCS, or the cluster of nodes connected only to each other. The percolation process ended when the LCCS had fewer than three nodes. Once the percolation process terminated, we calculated the percolation integral $\phi $ indicative of the area under the curve, representing the LCCS across all the percolation steps. Indeed, the LCCS is similarly defined as the sum of all LCCS weighted by their weight threshold value (31). In this study, we implemented percolation analysis using the weighted unfiltered networks previously constructed from the WAT data. According to Kenett and colleagues (31), we control for the fluency bias (i.e., numerous answers were possible for each target word) estimating the ratio between the average amount of responses for each condition. Therefore, we normalized the weights of the RA network by a factor of 2.20 (the ratio between the average amount of responses to RA and CR conditions) and the weights of OR network by a factor of 1.67 (the ratio between the average amount of responses of OR and CR conditions). See Fig. S1 in Supplementary Materials for a comparison of the distribution of link strengths in both networks.

We then used four approaches to examine the statistical significance of our findings, namely LOSO, LONO, the link shuffling, and the effect of noise analyses. In accordance with the previous analysis, to conduct the LONO and LOSO procedures, we iteratively excluded one node or one participant, respectively. Then the percolation procedure was run on the resulted partial networks, computing the percolation integral $\phi $. Next, the Link Shuffling analysis was conducted in order to run out the case that differences between networks were due to a difference in link weights. This was done by randomly selecting two pairs of nodes and exchanging their links in the network. For example, given a pair of nodes a and b with link strength 0.5 and a pair of nodes c and d with link strength 0.7 we exchanged edge weight such that a and b are connected with 0.7 and c and d with 0.5. For each link in each network, this process was repeated 10 times, aiming to exchange the majority of the links (∼ 1140 shuffles). This procedure was repeated with 500 iterations, computing $\phi $ on the link-shuffled network at each iteration. Lastly, we implemented the noise analysis by adding noise to network link weights, using 500 iterations, and then computing the $\phi $. According to (31), in each iteration, Gaussian noise was added to the network links with a mean value of zero and a varied standard variation, of 10 to 4 to 10 to 3.

We then conducted permutation *t* tests ($\alpha $ = 0.05,10,000 iterations) between the $\phi $ of the CR, OR, and RA conditions for each procedure described above. We corrected for multiple comparisons using Bonferroni correction ($\alpha $ = 0.017).

### Distributional semantics analysis

WAT data are also analyzed using a distributional semantics approach, which is based on the assumption that semantically similar words tend to appear in similar contexts (36). We extracted word embeddings (vectorial representations of words) for each target, category, and response word using pretrained word2vec models (66). We decided to use word2vec models and not transformers like models, such as BERT, since our task did not require to model the semantic context and also because the superiority of transformers models with respect to word2vec models is not established in the literature (89). These models come mostly in two versions, called continuous bag Of words (CBOW) and Skip-gram. Both models are deep neural networks (DNN) that employ a sliding window approach to move through text corpora. CBOW is trained to maximize the probability of the target word by looking at the context, while Skip-gram is trained to predict the context from a target word (90). The context is represented as a bag of words contained in a fixed size window around the target word. A vector representation of each word in the corpus is extracted from the hidden layer as a result of the DNN training and used as the word embedding values. Word2vec models have already been shown to correlate highly with judgments of human relatedness (67) as well as human creativity ratings (). For methodological robustness, in this study, we used three pretrained models trained with different methods (CBOW or Skip-gram) and different Italian text corpora. The first model was trained with the CBOW method using fastText on a concatenation of the Common Crawl and Wikipedia (91). The second model was trained with the Skip-gram method on Wikipedia (92). The third model was trained with the Skip-gram method using fastText on the OpenSubtiles corpus (93). All models were trained with a window size of 5, 10 negatives, and an embedding dimensionality of 300 (for further details, see their respective references). We extracted word embeddings of the target, category, and participants’ preprocessed response words (see above) from all the models and averaged them. We opted for this approach since it reduces the influence of a specific model while increasing the reliability of the results (67). In the rare case that words did not have word embeddings for a specific model (less than 1%), we averaged word embeddings from the available models (). Then, for each target-response pair, we computed semantic similarity as the cosine angle between a target and response embedding vectors, and subtracting it to 1 to obtain a semantic distance index, as follows

(1) $$\begin{eqnarray*}
\mathcal{S}\mathcal{D}\ \left( {x,y} \right) = {\rm{\ }}1 - \frac{{\mathop \sum \nolimits_{i\ = {\rm{\ }}1}^n {x}_i{y}_i}}{{\sqrt {\mathop \sum \nolimits_{i\ = {\rm{\ }}1}^n x_i^2} \sqrt {\mathop \sum \nolimits_{i\ = {\rm{\ }}1}^n y_i^2} }},
\end{eqnarray*}$$

where $\mathcal{S}\mathcal{D}$ is the semantic distance function, *x* and *y* are the word embeddings and *n* is the dimensionality. Averaged semantic distance scores were then calculated across participants for each condition. Furthermore, we defined a category switching index by computing the semantic distance between a target word and all the selected category words as well as between the response of that target and the categories. Therefore, we assigned a category to the target and the response words as the one that has the minimum semantic distance, as follows

(2) $$\begin{eqnarray*}
{\rm{\ }}\mathcal{z}_j = \ \mathcal{S}\mathcal{D}\left( {x,{k}^j} \right),
\end{eqnarray*}$$

(3) $$\begin{eqnarray*}
c\ = \mathop {{\rm{argmin}}}\limits_{\mathcal{z}} \mathcal{z},
\end{eqnarray*}$$

where *x* can be either the word embedding of the target or the response, ${k}^j$ is the *j*-the category word embedding, $\mathcal{z}$ is the vector of semantic distances between either the target or the response and the categories and ${\boldsymbol{c}}$ is the index of the corresponding category. The category switching index was then computed as 1 minus the Kronecker delta function applied to the target category index and the response category index, as follows

(4) $$\begin{eqnarray*}
\mathcal{C}\mathcal{S}\ \left( {{c}_t,{c}_r} \right) = {\rm{\ }}1 - \{ \begin{array}{@{}*{1}{c}@{}} {0{\rm{\ }}if{\rm{\ }}{c}_t \ne {c}_r}\\ {1{\rm{\ }}if{\rm{\ }}{c}_t = {c}_r\ } \end{array},
\end{eqnarray*}$$

where $\mathcal{C}\mathcal{S}$ is the category switching function, ${c}_t$ and ${c}_r$ are, respectively, the target and response category index. For statistical comparison, we averaged semantic distance and category switching values across target and responses for each participant and condition. Two-tails paired permutation *t* tests ($\alpha $ = 0.05,10,000 iterations) were performed between the values of the CR, OR, and RA conditions for semantic distance and category switching. We corrected for multiple comparisons using Bonferroni correction (α_Bonferroni_ = 0.017). In order to examine the relations between the three model spaces and the average one, we conducted a Pearson correlation analysis on the semantic distances computed with each model. The results revealed a positive significant correlation among all pairs (all r > 0.83, *P* < 0.001; see Table S6 in Supplementary Materials). Finally, in order to visualize the responses in the embedding space (Fig. 4a), we used the t-SNE algorithm to reduce the dimensionality of the word embeddings from 300 to 3, using principal component analysis to initialize the embedding space, a perplexity value of 50, early exaggeration of 12, learning rate of 200,1000 iterations and cosine distance as distance metric (94). As for the previous network analysis, we controlled for possible associative fluency bias. Thus, we ran the distributional analysis using only the first two associative responses given by each participant to a target word (33), analyzing differences between conditions.

### Computational modeling analysis

Finally, we implemented a computational modeling analysis to gain insight into the computational mechanisms underlying the semantic navigation strategies adopted by the participants. All the analysis steps were carried out in Python with the Tensorflow library (95). For this, we proposed a computational model, which we called the SemExp, that consists of two components. The first component is a similarity matrix between target words (40 in our study) and all the unique responses given by the whole sample to every condition (here, 3,328). This matrix (40 × 3,328) is created from the averaged word embeddings previously extracted from the word2vec models (Fig. 5a) and can be seen as an approximation of a value function operating in a semantic state space (59). The entries of this matrix are similarity scores computed as the inverse of the semantic distance score computed as in eq.1. We decided to compute the similarity score in this way, since we wanted to have nonnegative values and the cosine similarity ranges also toward negative values. The second component of the model is an action selection rule to generate associative responses given a target word. This response generation is performed by selecting the vector of similarity values of a given target word and the computing the softmax function ($\sigma $) on this vector as follows

(5) $$\begin{eqnarray*}
{\sigma }_i\ \left( x \right) = \frac{{exp\left( {\beta {x}_i} \right)}}{{\mathop \sum \nolimits_j exp\left( {\beta {x}_j} \right)}},
\end{eqnarray*}$$

where *x* is the vector of similarity scores of a target word, $exp$ is the exponential function and $\beta $ is the inverse temperature parameter that controls the amount of exploration vs. exploitation. Basically, this parameter determines the degree to which the value function affects choice. In other words, it controls the level of randomness in the selection of the associative response, where lower values of *β* induce the model to stochastically generate responses (i.e,. *β*<1 indicates more exploratory behavior) and higher *β* permits the model to deterministically select the most probable response word (i.e., *β*>1 indicates more exploitative behavior). We performed model fitting using the NLL as the loss function, also called cross-entropy loss, as follows

(6) $$\begin{eqnarray*}
NLL\ \left( {y,\hat{y}} \right) = {\rm{\ }} - \mathop \sum \limits_{i\ = {\rm{\ }}1}^k {y}_i{\rm{\ }}log\left( {\widehat {{y}_i}} \right),
\end{eqnarray*}$$

where *y* is the one-hot encoded vector of response given by a certain participant to a target word in a certain condition, $\hat{y}$ is the model prediction (i.e., the output of the softmax function) and *k* is the dimensionality of the vectors, which in our case is 3,328. To perform the parameter estimation procedure, we used the gradient descent algorithm since our model was fully differentiable. Thus, for each participant and condition, we stacked all responses to every target word and let the model predict them in a single forward pass, given as input the index of the target words. Then, we updated the *β* parameter as following

(7) $$\begin{eqnarray*}
{\rm{\beta \ }}\colon {\rm{\ \beta }} - {\rm{\ \alpha }}\frac{{\partial NLL}}{{\partial {\rm{\beta }}}},
\end{eqnarray*}$$

where $\alpha $ is the learning rate that we set to 0.01 and $\frac{{\partial NLL}}{{\partial {\rm{\beta }}}}$ is the partial derivative of the loss function NLL with respect to the *β* parameter. In order to obtain a reliable estimation, we ran the optimization procedure 10 times. For each run, we initialized the *β* parameter by sampling it uniformly in the range [0.5 to 5] and let the optimization procedure running for a maximum number of iterations equal to 10,000. On each iteration, we checked the convergence of the procedure defined as the difference between consecutive loss values being less than a tolerance parameter, here set to ${10}^{ - 7}$, and a patience parameter of 20 iterations. At the end of this procedure, we selected the *β* parameter as the one with the lowest NLL value. Before fitting the SemExp model to the participants’ choices, we performed a parameter recovery analysis to check whether the amount of data collected was enough for our parameter estimation procedure. For this, we generated artificial data using the SemExp model with the same amount of data collected per participant and condition and the true *β* parameter sampled uniformly in the range [0.1 to 4]. Then we fitted the model on the artificial data using the optimization procedure described above and computed the Pearson correlation coefficient between the fitted and real parameters. Next, we performed model selection by comparing three models. The first was a null model consisting of an uniform probability distribution for generating responses. In other words, regardless the target word, the probability of generating every response word was ${k}^{ - 1}$. The second was a fixed model (WordEmb) consisting only of the first component of the SemExp model, which is the similarity matrix built on the averaged word embeddings. The third was the SemExp model with the free parameter β fitted for each participant and condition. To visually inspect the loss landscape of the SemExp model, we also ran a grid search procedure by computing the values of the NLL loss as a function of the *β* parameter, linearly sampled in a range from 0.1 to 5 with 50 steps for each participant and condition. Statistical analysis comparing the models’ NLL values and the *β* parameters across conditions was carried out using two-tails paired permutation *t* tests ($\alpha $ = 0.05,10,000 iterations), corrected for multiple comparisons using Bonferroni correction (${a}_{Bonferroni}$ = 0.017).

## Supplementary Material

pgac273_Supplemental_File

## Data Availability

The datasets generated and analyzed during the current study are not publicly available to comply with the EU's General Data Protection Regulations 2018 (GPDR), since participants did not provide explicit written consent regarding the sharing of their data on public repositories. Researchers interested in the data need to contact the corresponding authors and provide a research plan for using the data. This plan will be submitted to the ethical committee to obtain access to the data. Source data regarding all the results and statistics displayed in the tables and figures are provided at the following link: https://osf.io/vumf3/. Regarding the code, all the analyses were carried out using R (version 4.1.1) and Python (version 3.7). Semantic network analyses were carried out using the following publicly available libraries: - The SemNeT package (1.4.4). https://github.com/AlexChristensen/SemNeT - The NetworkToolbox package (1.4.2). https://cran.r-project.org/web/packages/NetworkToolbox/index.html - The NetworkX package (2.8.6). https://networkx.org/ Distributional semantics and computational modeling analyses were carried out using the following publicly available libraries: - The Scipy package (1.9.0). https://scipy.org/ - The Tensorflow package (2.5). https://www.tensorflow.org/ The word2vec deep learning models utilized in this study are available at the following links: - The first model was trained with the CBOW on italian Common Crawl and Wikipedia using fastText. https://fasttext.cc./docs/en/crawl-vectors.html - The second model was trained with the Skip-gram method on italian Wikipedia. https://wikipedia2vec.github.io/wikipedia2vec/pretrained/ - The third model was trained with the Skip-gram method using fastText on the Italian OpenSubtiles corpus. https://github.com/jvparidon/subs2vec

## Notes

**Classification:** Psychological and Cognitive Sciences
