# Supplementary material for: Balancing novelty and appropriateness leads to creative associations in children
Source: PNAS Nexus. 2022 Dec 2;1(5):pgac273. doi: 10.1093/pnasnexus/pgac273 (PMC9802071; doi:10.1093/pnasnexus/pgac273)
Supplement: pgac273_Supplemental_File [file pgac273_supplemental_file.docx]

Balancing novelty and appropriateness leads to creative associations in children

Rastelli C., Greco A., De Pisapia N., Finocchiaro C.

**Supplementary Information**

**Stimuli Selection**

Semantic categories were selected a priori from a list of 32 Italian production norms gathered by (1) and accordingly to the most commonly used categories in the cognitive neuroscience literature (2–4). Target words were taken from the Lexvar database (5). Here, in order to facilitate lexical retrieval and concept representation, linguistic features were ordered sorting the highest values for imageability, familiarity, and concreteness. Moreover, words were controlled by applying a fixed range for length in syllables (2:4) and letters (4:8). The first four words for each category were extracted, excluding ambiguous words such as “color”. The final set of stimuli used in the experiments consists of 40 words from 10 concept categories; see Table S1.

**Table S1.** Word stimuli set.

| **Domain** | **Category** | **Target Words** |
| --- | --- | --- |
| Natural | Person | Madre; Padre; Nonno; Figlio (*Mother; Father; Grandfather; Son*) |
|  | Body Parts | Mano; Bocca; Naso; Testa (*Hand; Mouth; Nose; Head*) |
|  | Animals | Cane; Gatto; Cavallo; Coniglio (*Dog; Cat; Horse; Rabbit*) |
|  | Fruit | Mela; Limone; Banana; Castagna (*Apple; Lemon; Banana; Chestnut*) |
|  | Nature | Acqua; Pietra; Fuoco; Sabbia (*Water; Stone; Fire; Sand*) |
| Artefact | Furniture | Tavolo; Sedia; Poltrona; Vasca (*Table; Chair; Sofa; Basin*) |
|  | Stationery | Libro; Matita; Quadreno; Vernice (*Book; Pencil; Textbook; Paint*) |
|  | Food | Pane; Torta; Sugo; Zuppa (*Bread; Pie; Sauce; Soup*) |
|  | Vehicles | Auto;Treno; Nave; Furgone (*Car; Train; Ship; Van*) |
|  | Urban Buildings | Scuola; Negozio; Ospedale; Palestra (*School; Shop; Hospital; Gym*) |

**Word Association Task (WAT): Instruction set**

**Instruction for the “Ordinary” condition**

“Qui presentiamo istruzioni specifiche su come rispondere alla Fase 1. Ti verrà consegnata una lista di nomi, per ciascuno scrivi una o più parole che ti vengono in mente seguendo queste istruzioni: quando vedi la parola iniziale scrivi accanto una risposta usando una parola molto COMUNE. Per “comune” si intende una parola che è chiaramente collegata al nome e che viene usata spesso in associazione alla parola iniziale. Rispondi usando una parola che verrebbe probabilmente in mente a tutti quando leggono il nome di partenza”. *[EN: Here we present specific instructions for how to respond in Phase 1. You will be provided with a list of nouns, for each of them write one or more words that come to your mind following these instructions: when you see the initial noun write a response next to it using a very common word. By “common” we mean a word that is clearly related to the noun, and very often used in association with the noun. A word that would probably come to most everyone’s mind when they read the noun].*

**Instruction for the “Random” condition**

Qui presentiamo istruzioni specifiche su come rispondere alla Fase 2. Nota bene che queste istruzioni sono diverse dalla Fase 1. Ti verrà consegnata una lista di nomi, per ciascuno scrivi uno o più nomi che ti vengono in mente seguendo queste istruzioni: Quando vedi la parola iniziale scrivi accanto una risposta usando una parola molto INSOLITA. Per “insolita” si intende una parola che non è collegata con il nome iniziale. Una parola che normalmente non verrebbe in mente a nessuno quando legge il nome di partenza. *[EN: Here we present specific instructions for how to respond in Phase 2. Notice that these instructions are different than Phase 1. You will be provided with a list of nouns, for each of them write one or more words that come to your mind following these instructions: give a very unusual word response to the noun. By “unusual” we mean a word that is unrelated to the noun. A word that would probably not come to anyone else’s mind when they read the noun].*

**Instruction for the “Creative” condition**

Qui presentiamo istruzioni specifiche su come rispondere alla Fase 3. Nota che queste istruzioni sono diverse dalle Fasi 1 e 2. Ti verrà consegnata una lista di nomi, per ciascuno scrivi uno o più nomi che ti vengono in mente seguendo queste istruzioni: quando vedi la parola iniziale scrivi accanto una risposta usando una parola molto CREATIVA. Per “creativa” si intende una parola che è chiaramente collegata con il nome inziale ma che si usa raramente. Una parola che probabilmente verrebbe in mente a pochissime altre persone. *[Here we present specific instructions for how to respond in Phase 3. Notice that these instructions are different than Phases 1 and 2. You will be provided with a list of nouns, for each of them write one or more words that come to your mind following these instructions: give a very creative or original verb response to the noun. By “creative”, we mean a word that is clearly related to the noun, and also rarely used in association with the noun. A verb that would probably come to mind for very few other people].*

**Human ratings**

**Table S2.** Descriptive statistics and inferential results from the paired two-tailed permutation t-test comparing human ratings for appropriateness, creativity and novelty of responses across condition.

|  | OR | CR | RA | CR vs. OR | | OR vs. RA | | CR vs. RA | |
| --- | --- | --- | --- | --- | --- | --- | --- | --- | --- |
|  | $M\left( SD \right)$ | $M\left( SD \right)$ | $M\left( SD \right)$ | $t$ | $d$ [ci 95%] | $t$ | $d$ [ci 95%] | $t$ | $d$ [ci 95%] |
| Appropriateness | 4.22 (0.26) | 3.91 (0.43) | 1.54 (0.33) | 5.287 | 0.83 [0.45, 1.22] | 45.711 | 8.86  [7.64, 10.07] | 31.847 | 6.08 [5.20, 6.95] |
| Creativity | 1.92 (0.13) | 2.08 (0.21) | 1.76 (0.12) | 5.34 | 0.92 [0.53, 1.31] | 7.331 | 1.17 [0.76, 1.75] | 10.282 | 1.81 [1.37, 2.25] |
| Novelty | 1.68 (0.20) | 1.98 (0.38) | 4.25 (0.33) | 6.289 | 0.98 [0.58, 1.37] | 47.433 | 9.26 [8, 10.53] | 33.511 | 6.25 [5.35, 7.15] |
| The t-statistics (from a parametric t-test student) and Cohen’s d (6) values are presented. Cohen’s d effect sizes: 0.20, small; 0.50, moderate; 0.80, large; 1.10, very large. All p <0.001. | | | | | | | | | |

**Semantic networks topology**

**Table S3.** Results from the paired two-tailed permutation t-test comparing conditions on the topology quantifiers of partial networks, built using only the first two responses of the WAT dataset.

|  | **OR** | **CR** | **RA** | **CR vs. OR** | | **OR vs. RA** | | **CR vs. RA** | |
| --- | --- | --- | --- | --- | --- | --- | --- | --- | --- |
| Meas | $M\left( SD \right)$ | $M\left( SD \right)$ | $M\left( SD \right)$ | $t$ | $d$ [ci 95%] | $t$ | $d$ [ci 95%] | $t$ | $d$ [ci 95%] |
| **Full Network** | | | | | | | | | |
| ASPL | 3.14 | 2.82 | 2.35 |  |  |  |  |  |  |
| CC | 0.52 | 0.49 | 0.40 |  |  |  |  |  |  |
| Q | 0.59 | 0.54 | 0.43 |  |  |  |  |  |  |
| S | 2.63 | 2.85 | 2.66 |  |  |  |  |  |  |
| **LONO** | | | | | | | | | |
| ASPL | 3.09 (0.12) | 2.75 (0.09) | 2.36 (0.07) | -15.12 | 3.18 [2.52, 3.81] | 31.227 | 7.2  [6, 8.40] | 21.118 | 4.88 [4.01, 5.75] |
| CC | 0.52 (0.01) | 0.48 (0.01) | 0.41 (0.02) | -13.49 | 2.83 [2.21, 3.45] | 31.635 | 7.61 [6.35, 8.87] | 20.812 | 5.05 [4.16, 5.95] |
| Q | 0.58 (0.01) | 0.53 (0.01) | 0.44 (0.02) | -19.444 | 3.92 [3.17, 4.67] | 29.337 | 6.92 [5.76, 8.08] | 20.284 | 4.69 [3.08, 4.55] |
| S | 2.60 (0.07) | 2.78 (0.04) | 2.65 (0.03) | 13.392 | 3.39 [2.70, 4.07] | -4.125 | 0.98 [0.52, 1.45] | 17.46 | 3.82 [3.08, 4.55] |
| **LOSO** | | | | | | | | | |
| ASPL | 3.15 (0.03) | 2.72 (0.12) | 2.36 (0.09) | -26.345 | 5 [4.25, 5.75] | 63.082 | 12.1 [10.47, 13.73] | 18.966 | 3.53 [2.93, 4.12] |
| CC | 0.52 (0.01) | 0.48 (0.02) | 0.40 (0.01) | -20.575 | 3.75 [3.13, 4.36] | 57.773 | 10.51 [9.08, 11.93] | 24.829 | 4.8  [4.07, 5.52] |
| Q | 0.59 (0.01) | 0.53 (0.01) | 0.45 (0.02) | -32.997 | 6.11 [5.23, 6.99] | 52.362 | 9.75 [8.42, 11.08] | 22.656 | 4.54 [3.84, 5.24] |
| S | 2.66 (0.02) | 2.83 (0.04) | 2.70 (0.04) | 25.934 | 5.13 [4.36, 5.90] | -6.751 | 1.25 [0.85, 1.66] | 21.053 | 3.49 [2.90, 4.08] |
| ASPL = Average Shortest Path Length; CC = Clustering Coefficient; Q = modularity index; S = smallworld-ness. LONO = Leave-One-Node-Out; LOSO = Leave-One-Subject-Out. The t-statistics (from a parametric t-test student) and Cohen’s d (6) values are presented. Cohen’s d effect sizes: 0.20, small; 0.50, moderate; 0.80, large; 1.10, very large. All p <0.0001, corrected for multiple comparisons using Bonferroni (0.017). | | | | | | | | | |

**Network Percolation**

**Figure S1.** Comparison of the distribution of link strengths in condition based-networks.


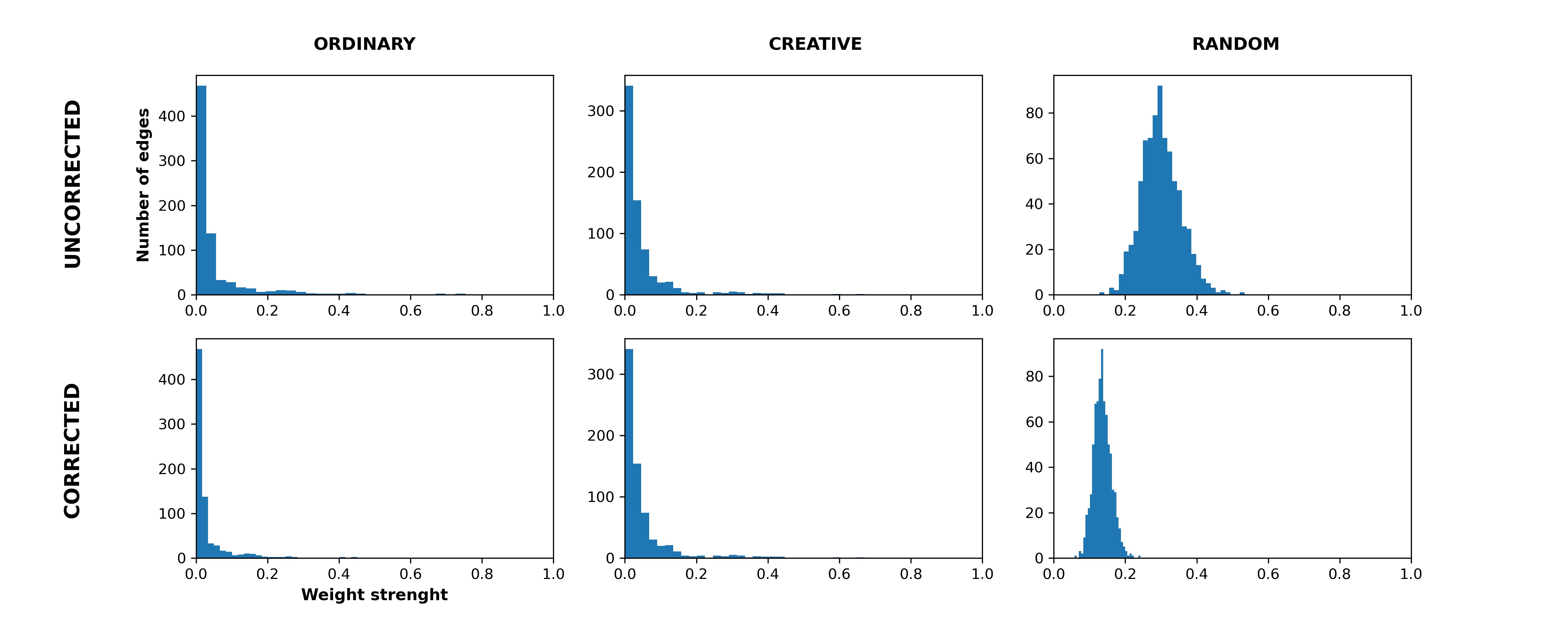


**Semantic distance and category switching**

**Table S4.** Descriptive statistics for semantic distance and category switching considering the first two responses of the WAT dataset.

|  | OR | CR | RA |
| --- | --- | --- | --- |
|  | $M\left( SD \right)$ | $M\left( SD \right)$ | $M\left( SD \right)$ |
| Semantic Distance (*SD*) | 0.64 (0.03) | 0.66 (0.02) | 0.77 (0.03) |
| Category Switching (*CS*) | 0.64 (0.08) | 0.68 (0.07) | 0.87 (0.06) |

**Table S5.** Results from conditions comparisons using the two-tailed paired-sample permutation t-test on semantic distance and category switching considering the first two responses of the WAT dataset.

|  | CR vs. OR | | | OR vs. RA | | | CR vs. RA | | |
| --- | --- | --- | --- | --- | --- | --- | --- | --- | --- |
|  | $t$ | $p$ | $d$ [ci 95%] | $t$ | $p$ | $d$ [ci 95%] | $t$ | $p$ | $d$ [ci 95%] |
| *SD* | 3.549 | <0.0001 | 0.62 [0.24, 1] | 20.643 | < 0.0001 | 4.49 [3.8, 5.19] | 22.858 | < 0.0001 | 4.31 [3.63, 4.98] |
| *CS* | 3.014 | 0.004 | 0.50 [0.12, 0.88] | 15.293 | < 0.0001 | 3.2 [2.64, 3.76] | 15.177 | < 0.0001 | 2.91 [2.38, 3.45] |
| Results from the paired two-tailed permutation t-test on semantic distance (SD) and category switching (CS) comparing the CR, OR and RA conditions. T-statistics estimated from a parametric t-test. Cohen’s $\boldsymbol{d}$ effect sizes: 0.20, small; 0.50, moderate; 0.80, large; 1.10, very large. All p-values were corrected for multiple comparisons using Bonferroni (0.017). | | | | | | | | | |

**Table S6.** Descriptive statistics and correlations between the semantic distance predictive models and their averaged index.

|  | **Semd_m1** | **Semd_m2** | **Semd_m3** |
| --- | --- | --- | --- |
| Semd_CC_wiki | - |  |  |
| Semd_Wiki | .83 *** | - |  |
| Semd_sub | .85 *** | .86 *** | - |
| Semd_avg | .94 *** | .94 *** | .96 *** |
| Note. (***) p = < 0.001. Semd_CC_wiki = concatenation of the Common Crawl and Wikipedia; Semd_Wiki = Wikipedia; Semd_sub = OpenSubtiles. | | | |

References

1. M. Montefinese, E. Ambrosini, B. Fairfield, N. Mammarella, Semantic memory: A feature-based analysis and new norms for Italian. *Behavior Research Methods* **45**, 440–461 (2013).

2. S. De Deyne, D. J. Navarro, A. Perfors, M. Brysbaert, G. Storms, Measuring the associative structure of English: The “Small World of Words” norms for word association. 1–26 (2016).

3. G. Kremer, M. Baroni, A set of semantic norms for German and Italian. *Behavior Research Methods* **43**, 97–109 (2011).

4. K. McRae, G. S. Cree, M. S. Seidenberg, C. Mcnorgan, Semantic feature production norms for a large set of living and nonliving things. *Behavior Research Methods* **37**, 547–559 (2005).

5. L. Barca, L. S. Arduino, C. V. Marinelli, C. Burani, Lexical and sublexical variables_2: norms for 626 Italian nouns (2015).

6. J. Cohen, A power primer. *Psychological bulletin* **112**, 155 (1992).
